# Shedding the Light on *Litopenaeus vannamei* Differential Muscle and Hepatopancreas Immune Responses in White Spot Syndrome Virus (WSSV) Exposure

**DOI:** 10.3390/genes11070805

**Published:** 2020-07-16

**Authors:** Camilla A. Santos, Sónia C. S. Andrade, Jorge M. O. Fernandes, Patrícia D. Freitas

**Affiliations:** 1 Departamento de Genética e Evolução, Universidade Federal de São Carlos, São Carlos 676, Brazil; 2 Departamento de Genética e Biologia Evolutiva, Instituto de Biociências, Universidade de São Paulo, São Paulo 05508-090, Brazil; soniacsandrade@ib.usp.br; 3 Faculty of Biosciences and Aquaculture, Nord University, 8026 Bodø, Norway; jorge.m.fernandes@nord.no

**Keywords:** innate immunity, RNA-seq, differential expression, WSSV resistance, shrimp

## Abstract

White Spot Syndrome Virus (WSSV) is one of the main threats to farming *Litopenaeus vannamei*, the most important crustacean commercialized in aquaculture worldwide. Here, we performed RNA-seq analyses in hepatopancreas and muscle from WSSV-negative (healthy) and WSSV-positive (unhealthy) *L. vannamei,* previously exposed to the virus, to obtain new insights about the molecular basis of resistance to WSSV. We detected 71% of our reads mapped against the recently described *L. vannamei* genome. This is the first report mapping RNA-seq transcripts from shrimps exposed to WSSV against the species reference genome. Differentially expressed gene (DEG) analyses were performed for four independent comparisons, and 13,338 DEGs were identified. When the redundancies and isoforms were disregarded, we observed 8351 and 6514 DEGs, respectively. Interestingly, after crossing the data, we detected a common set of DEGs for hepatopancreas and healthy shrimps, as well as another one for muscle and unhealthy shrimps. Our findings indicate that genes related to apoptosis, melanization, and the Imd pathway are likely to be involved in response to WSSV, offering knowledge about WSSV defense in shrimps exposed to the virus but not infected. These data present potential to be applied in further genetic studies in penaeids and other farmed shrimp species.

## 1. Introduction

Marine shrimp farming is a worldwide profitable activity, with special emphasis on *Litopenaeus vannamei*, a species from the western Pacific Ocean which presents excellent breeding rates in captivity [1,2,3]. Despite this penaeid being one of the most commercialized shrimps in the world, the high incidence of pathogens in aquaculture, and also in nature, is a constant concern to growth and survival of this species. Pathogens, such as White Spot Syndrome Virus (WSSV) [4,5], Taura Syndrome Virus (TSV) [6,7], and Yellow Head Virus (YHV) [8], spread quickly in the aquatic environment and even more frequently in captivity.

Arthropod immunity is based on cellular and immune responses to cope with pathogens and external stimuli [9,10]. In an attempt to combat pathogens, the organism undergoes strong metabolic stress due to the production of reactive oxygen species (ROS), which can cause damage to cellular and DNA structures [11,12]. In spite of the description of some proteins acting on decapod immunity and some of the strategies adopted against pathogens [11,13,14,15,16], the processes involved in crustacean immunology are not elucidated yet, and the development of disease control methods is still limited. Among the current prophylaxis strategies applied to avoid infections in shrimp are the injections of specific virus sequences, which enable an improved immune response by acting analogously to interferons in mammals [17,18]. Similarly, approaches based on interference-RNAs (RNAi) have also been applied to provide sequence-specific injections to improve the response against some viral diseases, including WSSV [19,20]. However, an efficient treatment to combat this lethal pathogen is not available to date.

White spot disease (WSD) is highly harmful for shrimp, and may cause 100% mortality in farming conditions, resulting in enormous losses to shrimp market yearly [21]. The syndrome is caused by an enveloped double-stranded DNA virus of the *Nimaviridae* family, and the proteins contained in the envelope are known to be central for cell recognition, virus invasion and proliferation in the host [22]. Despite efforts to improve our understanding of immunity in decapods against WSSV [23] and other pathogens, such as TSV [7] and YHV [8], many immunological proteins and their respective mechanisms of action still remain unknown, making genetic and physiological studies related to these issues absolutely necessary. 

Transcriptomics is a powerful approach for the identification of genes and proteins related to the immune metabolism in shrimp [4,24]. In a recent RNA-seq study performed in *L. vannamei* gills, shrimps were injected with WSSV and differentially expressed gene analyses were performed in control and infected animals at different post-infection periods, showing that the expression patterns of genes related to the immune system were altered throughout the infection period [5].

In the present study, we used RNA-seq to identify differentially expressed genes (DEGs) in hepatopancreas and muscle of WSSV-negative and WSSV-positive *L. vannamei,* focusing on genes coding for proteins involved in the innate immunity in arthropods. Because the crustaceans have an open circulatory system, many of the immune proteins synthetized in the hemocytes are expressed in many body parts, including hepatopancreas and muscle. Hepatopancreas has a relevant role in immune defense in crustaceans, producing immune proteins, such as hemocyanin and lectin [25,26]. In addition, some of the most injured tissues by WSSV are the mesodermal ones, such as muscle [27].

As hypothesis, we considered that different gene expression profiles would be expected between WSSV-positive and WSSV-negative shrimps, and between hepatopancreas and muscle tissues. In this way, we sought for genes expressed in shrimps exposed to WSSV but not infected, aiming to identify key genes potentially related to virus defense, and to determine which of the tissues would be more active in this protection.

Our data showed an outstanding difference between the hepatopancreas and muscle expression profiles and a set of up-regulated genes with possible roles in resistance to WSSV infection. Further studies involving functional analyses of the genes highlighted herein as overexpressed in not-infected *L. vannamei* should verify the expression profiles obtained in response to the WSSV under different conditions. 

## 2. Material and Methods

### 2.1. Sampling and WSSV Exposure 

The *L. vannamei* samples used in this work were Specific Pathogen Free (SPF) post larvae (PLs) kept in a sanitary environment monitored for the presence of various pathogens, such as Infectious Hypodermal and Hematopoietic Necrosis Virus (IHHNV), Infection Myonecrosis Virus (IMNV), Taura Syndrome Virus (TSV), and WSSV. The PLs came from a single batch of a hybrid lineage developed by a Brazilian post larvae hatchery laboratory in Rio Grande do Norte state. First, we randomly sampled dozens of post larvae (PLs) in order to evaluate the presence of WSSV by qPCR.

The hemolymph was collected with a 1cc syringe, and 500 µL was used in qPCR, following recommendations [28,29]. The qPCR tests were carried out in a ABI 7500 Real-Time PCR equipment (Thermo Fisher Scientific, Waltham, MA, USA), using the primer pair set WSS1011F (5′-TGGTCCCGTCCTCATCTCAG-3′) and WSS1079R (5′-GCTGCTTGCCGGAAATTA-3′) [27], along with Platinum SYBR Green qPCR Super Mix UDG kit (Thermo Fisher Scientific, Waltham, MA, USA). 

After confirming the absence of the virus, thousands of PLs at about 45 days old (PL30) were released in an earthen pond of a Brazilian shrimp farm, with WSSV outbreaks, under standard husbandry conditions, using a pond aeration system [27]. Five days later, we sampled shrimps close to two months old (about 50 days old). The individuals were collected from a single fishing locally performed at the pond [30], and then they were evaluated morphologically for WSSV clinical signs, such as white spots in the carapace, reddish muscles, loose cuticle, and necrosis [27]. Ten symptomatic and ten asymptomatic shrimps were selected and individually diagnosed by qPCR again. For the WSSV diagnosis, the qPCR tests were performed as previously described [27,28,29]. 

For RNA-seq analyses, we collected hepatopancreas and muscle samples for both symptomatic and asymptomatic shrimps (Figure S1). The samples were stored in RNA later (Thermo Fisher Scientific, Waltham, MA, USA) and maintained at −80 °C. We also collected hemolymph for the RNA-seq approach, but, unfortunately, none of the samples yielded adequate amount of RNA for library construction. After the qPCR testes, only four symptomatic animals were detected as WSSV-positive. All asymptomatic shrimps were negative to the virus. We did not find any WSSV-positive asymptomatic shrimp. Results describing threshold cycle (Ct) values and viral loads are shown in Table S1.

### 2.2. Library Construction, Sequencing, and Trimming

cDNA libraries were constructed for muscle and hepatopancreas obtained from four WSSV-positive and four WSSV-negative shrimps. Thus, we constructed 16 transcriptome libraries for muscle and hepatopancreas from eight shrimps, as follows: (i) hepatopancreas of four WSSV-positive; (ii) muscle of four WSSV-positive; (iii) hepatopancreas of four WSSV-negative; and (iv) muscle of four WSSV-negative samples. These WSSV-positive samples were all symptomatic with WSSV clinical signs (unhealthy), whilst the WSSV-negative individuals were all asymptomatic for WSSV clinical signs (healthy). 

RNA isolation was performed using Chomczynski protocol [31], and then the 16 cDNA libraries were produced using the TruSeq RNA Library Preparation V2 kit (Illumina Inc., San Diego, CA, USA) [24]. All cDNA libraries were grouped in two flow cell lanes and sequenced on an Illumina HiSeq 2500 Platform (with 2 × 100 bp paired-end), using a TruSeq SBS V3 kit (Illumina Inc., San Diego, CA, USA). Each sample yielded one cDNA library. All reads are available at the Sequence Read Archive (SRA-NCBI) under the accession number SRP128934 (BioProject PRJNA428228). The quality of the raw data generated after sequencing was checked in the FastQC software (version 0.10.1) (http://www.bioinformatics.babraham.ac.uk/projects/fastqc/). The reads were filtered for Phred quality (QS) 23 (sequence average) and 30 (sequence edges), as well as minimum length of 65 bp, using the SeqyClean (v1.10.07) (https://github.com/ibest/seqyclean). 

### 2.3. Mapping and Differentially Expressed Genes Analyses

The reads were mapped against the *L. vannamei* reference genome (ASM378908v1) previously described for the species [32], using the software STAR version 2.7.0 [33]. Uniquely mapped read counts were used to determine differentially expressed genes (DEGs). For the DEGs identification, we performed four independent analyses as follow: hepatopancreas (Hp) versus muscle (Mu) tissues (Hp x Mu); healthy (He) versus unhealthy (Un) conditions, considering the tissue effect in a multi-factor analysis (He x Un); muscle of healthy (MuHe) versus muscle of unhealthy (MuUn) shrimps (MuHe x MuUn); and hepatopancreas of healthy (HpHe) versus hepatopancreas of unhealthy (HeUn) ones (HpHe x HeUn). In the comparison He x Un, between healthy and unhealthy animals, both hepatopancreas and muscle effects were considered in the analysis, and a multi-factor model with interaction was adopted.

For all the analyses, R/Bioconductor DEseq2 package [34] was used and normalization was performed by adjusting the distribution of the data according to a negative binomial distribution, followed by filtering the contigs by removing those with BaseMean < 5 considering all samples. The log2FC values and their respective *p*-values were calculated. Adjusted *p*-values were calculated using the Benjamini and Hochberg (BH) method [35]. Genes with False Discovery Rate (FDR) < 0.05 were considered significant for differential expression analyses, as follows: (Hp x Mu) log2FC < −0.5 up-regulated for hepatopancreas and log2FC > 0.5 up-regulated for muscle; (He x Un) log2FC < −0.5 up-regulated for healthy and log2FC > 0.5 up-regulated for unhealthy shrimps; (MuHe x MuUn) log2FC > 0.5 up-regulated for muscle of healthy and log2FC < −0.5 up-regulated for muscle of unhealthy shrimps; and (HpHe x HeUn) log2FC > 0.5 up-regulated for hepatopancreas of healthy and log2FC < −0.5 up-regulated for hepatopancreas of unhealthy shrimps.

With the purpose of identifying WSSV gene copies in exposed shrimps, we also mapped the transcriptome produced here against the WSSV genome (GenBank accession number ASM397254v1) [36], using Salmon software (v. 1.1.0) [37]. 

### 2.4. Functional Annotation and Enrichment in Gene Ontology (GO)

The functional annotation of the reference genome with protein-coding regions predicted by Transdecoder was performed in Trinotate [38] as a complementary approach to obtain a more complete and updated genome annotation. In the Trinotate pipeline, homologous sequences were queried in the Uniprot (uniref90 + SwissProt) [39], Kyoto Encyclopedia of Genes and Genomes (KEGG) [40], and Gene Ontology (GO) [41] databases, and later the ontologies Biological Process (BP), Molecular Function (MF), and Cell Component (CC) of GO were assigned. In order to identify the enriched GO terms in the set of genes differentially expressed against the reference genome in the two comparisons (Hp x Mu and He x Un), analyses were performed in the GOSeq package, version 1.24.0 [42] implemented in R. The enriched GO categories were calculated using the Wallenius [43] approximation and the *p*-values were adjusted using the BH method. The categories with adjusted *p*-value (FDR) < 0.1 were considered as enriched.

### 2.5. DEGs Protein-Protein Interactions (PPI) Networks

After DEGs analysis, those with log2FC ≥ 3 (absolute value) were used in the construction of the PPI (protein-protein interactions) networks to the identification of the most interactive genes. We used (i) 851, (ii) 1,005, (iii) 826, and (iv) 928 up-regulated genes for hepatopancreas and muscle tissues and healthy and unhealthy shrimps, respectively (Table S2). Their respective proteins were queried against the STRING (https://string-db.org) database for the identification of the experimental data and interactions among them. The PPI networks were visualized in Cytoscape software (v. 3.7.1) [44] and only the combined score value ≥ 0.4 and EdgeBetweenness ≥ 10 were considered for hub construction. The score is about the reliability of the protein interaction detected (higher the value, more reliable interactions) and the EdgeBetweenness algorithm relies on the connection strength between genes (higher values, stronger interactions). In the gene hub images, the node sizes are proportional to the number of connections observed for that protein (degree), and the edges thickness illustrates the interaction strength (EdgeBetweenness). The constructed hubs were submitted to enrichment analysis in Cytoscape, focusing on the immune/stress related proteins. 

## 3. Results

After processing and filtering the 16 sequenced libraries, we obtained 762,937,284 reads, of which 545,658,490 (71,5%) were mapped against the *L. vannamei* reference genome (Table S3). A total of 33,274 unigenes were annotated and used in the DEG analysis. From this total, (a) 12,899, (b) 8507, (c) 6979, and (d) 6773 returned hits, respectively, in SwissProt, GO, KEGG, and COG databases, with 5818 unigenes showing hits in all four databases (Figure S2).

**Figure 1:**
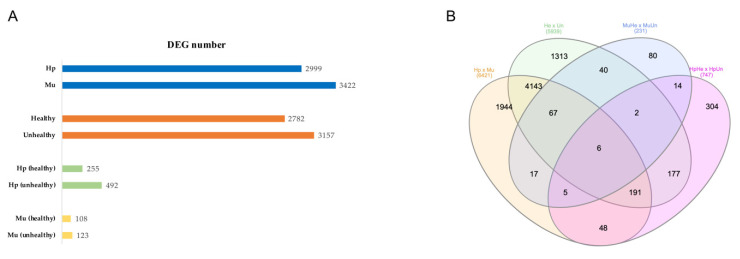
(**A**) Number of Differentially Expressed Genes (DEGs) detected for the four comparisons performed in the *Litopenaeus vannamei* transcriptome. (**B**) DEGs union. The total of 8351 unique DEGs from all the four analysis are represented by the sum of the values inside the Venn diagram. Hp x Mu: hepatopancreas (Hp) versus muscle (Mu); He x Un: healthy (He) versus unhealthy (Un), considering the tissue effect; MuHe x MuUn: muscle of healthy (MuHe) versus muscle of unhealthy (MuUn); and HpHe x HpUn: hepatopancreas of healthy (HpHe) versus hepatopancreas of unhealthy (HpUn) shrimps. WSSV: White Spot Syndrome Virus.

The comparison between tissues (Hp x Mu) showed an elevated number of DEGs highly up-regulated and distinct expression patterns between tissues, as expected. However, the multi-factor analysis generated the most informative data, revealing the determinant role of tissues in the expression profiles of healthy and unhealthy shrimps when exposed to WSSV and evidencing the greater number of DEGs with higher expression values (log2FC). The remaining analyses, considering hepatopancreas of healthy versus hepatopancreas of unhealthy (HpHe x HpUn), as well as muscle of healthy versus muscle of unhealthy (MuHe x MuUn) shrimps, showed a smaller number of DEGs (Figure 1 and Table S4). 

In total, we detected 13,338 DEGs for the four analyses performed, as follows: 6421 for Hp x Mu comparison, with 2999 DEGs observed for hepatopancreas (Hp) and 3422 for muscle (Mu); 5939 for He x Un, with 2782 for healthy (He) and 3157 for unhealthy shrimps (Un); 231 for MuHe x MuUn, with 108 for muscle of healthy (MuHe) and 123 for muscle of unhealthy (MuUn) animals; and 747 for HpHe x HeUn comparison, with 255 DEGs observed for hepatopancreas of healthy (HpHe) and 492 for hepatopancreas of unhealthy (HpUn) shrimps. Six common DEGs were found in all comparisons, and 3689 DEGs were not common among the analyses as shown in the Venn diagram (Figure 1B). When the redundancies and isoforms were disregarded, we observed 8351 and 6514 unique DEGs, respectively.

Regarding the reads mapped against the WSSV genome, 1,018,000 reads from both hepatopancreas and muscle tissues of unhealthy shrimps only (samples 17 and 4E), matched the WSSV genome (0.13%). Four DEGs were identified up-regulated in these unhealthy shrimps, two of them with known gene products: immediate-early protein and ribonucleotide reductase small subunit protein (Table S5). More details about the main findings are presented below.

### 3.1. Differentially Expressed Genes between Hepatopancreas and Muscle (Hp x Mu) Tissues

**Figure 2:**
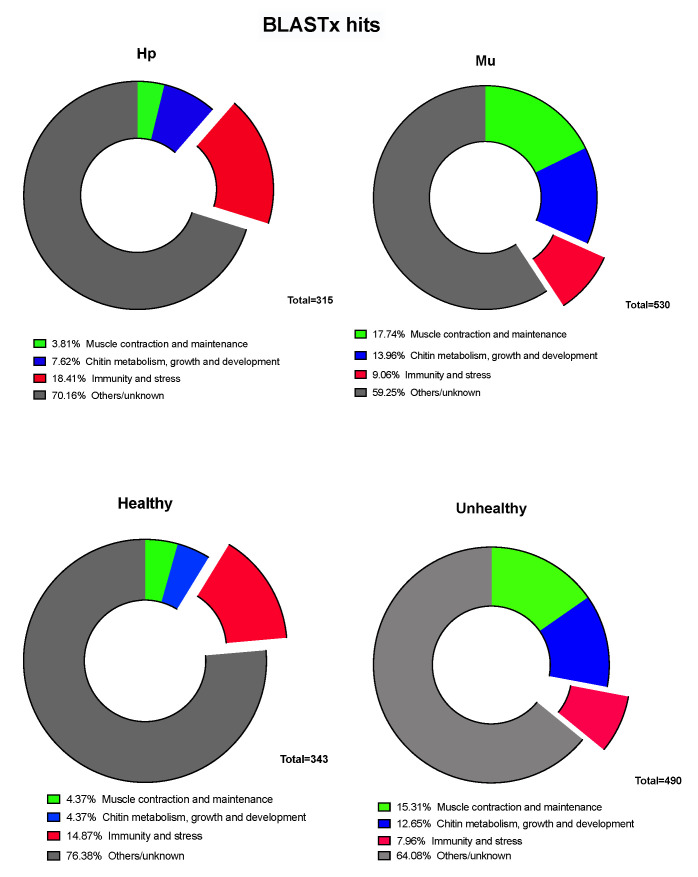
The most expressed genes identified in the transcriptome of hepatopancreas (Hp) and muscle (Mu) of WSSV-negative (healthy) and WSSV-positive (Unhealthy) *Litopenaeus vannamei* shrimps, with BLASTx hits in arthropod species. The part highlighted in light red represents the immune and stress genes. WSSV: White Spot Syndrome Virus.

**Figure 3:**
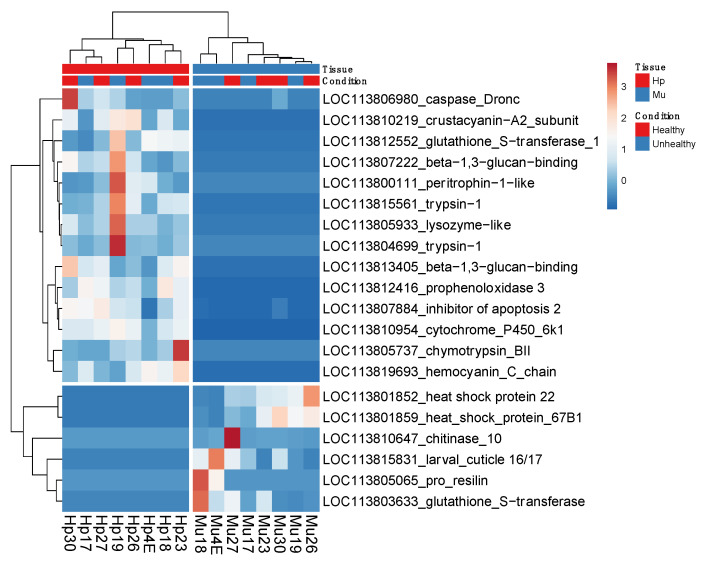
The most differentially expressed genes in common for hepatopancreas (Hp) and WSSV-negative (Healthy); and muscle (Mu) and WSSV-positive (Unhealthy) *Litopenaeus vannamei* shrimps. Unit variance scaling is applied to rows. Both rows and columns are clustered using correlation distance and average linkage. The numbers 0, 1, 2, and 3 in the color scale represent the normalization z-score values. The counts per million normalized reveals the clear difference in Hp and Mu expression patterns represented in a heatmap. The samples from these tissues are grouped separately. Intense red rectangles represent the samples with greater number of read counts for a particular gene. WSSV: White Spot Syndrome Virus.

**Figure 4:**
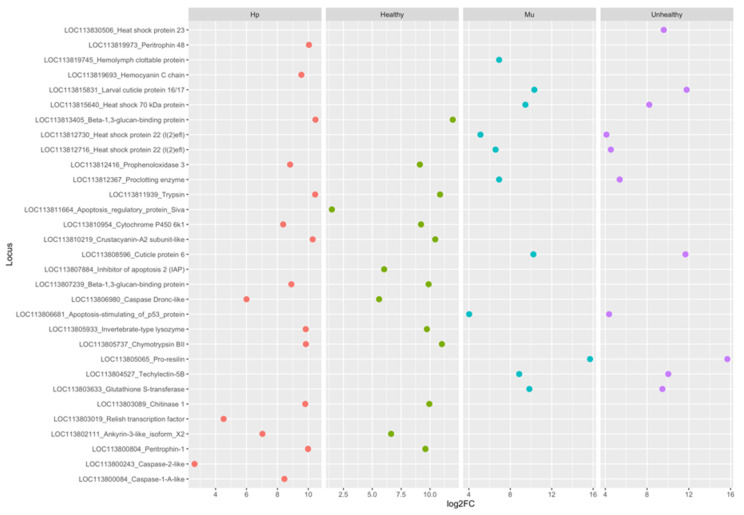
The main DEGs identified in the hepatopancreas (Hp) of WSSV-negative (Healthy) and in the muscle (Mu) of WSSV-negative (Unhealthy) *Litopenaeus vannamei* shrimps, with their respective log2FC values. WSSV: White Spot Syndrome Virus.

For the comparisons between tissues only, we identified 6421 DEGs (False Discovery Rate (FDR) < 0.05). From these, 2999 (46%) were up-regulated in hepatopancreas (log2FC < −0.5) and 3422 (54%) in muscle (log2FC > 0.5) (Figure S3 and Table S4). The up-regulated genes for hepatopancreas were down regulated for muscle and vice versa. When we considered the up-regulated genes with arthropod hits, we found 18.4% and 9% of gene products related to immunity and stress for hepatopancreas and muscle, respectively (Figure 2). The Euclidean distances between the expression values of the paired tissues were illustrated in a heatmap that showed muscle and hepatopancreas completely separated into distinct clades, indicating a clear and large difference between their genetic expression profiles (Figure S4). Among the most up-regulated genes in hepatopancreas involved in immunity responses in arthropods, we identified trypsin, peritrophin, β-1,3-glucan-binding, prophenoloxidase 3 (proPO3), lysozyme, and crustacyanin. On the other hand, in muscle, we found heat shock proteins (HSPs), chitinase 10, glutathione S-transferase, and pro-resilin protein genes among the most expressed ones (Figure 3 and Figure 4).

The GO terms identified for the hepatopancreas and muscle comparisons varied widely (Figure S5 and Table S6). Functional enrichment analysis of DEGs in GO showed 171 and 34 enriched GO terms (FDR < 0.1) for hepatopancreas and muscle, respectively (Table S7). 

### 3.2. Multi-Factor Analysis of Differentially Expressed Genes between Healthy and Unhealthy (He x Un) Conditions

**Figure 5:**
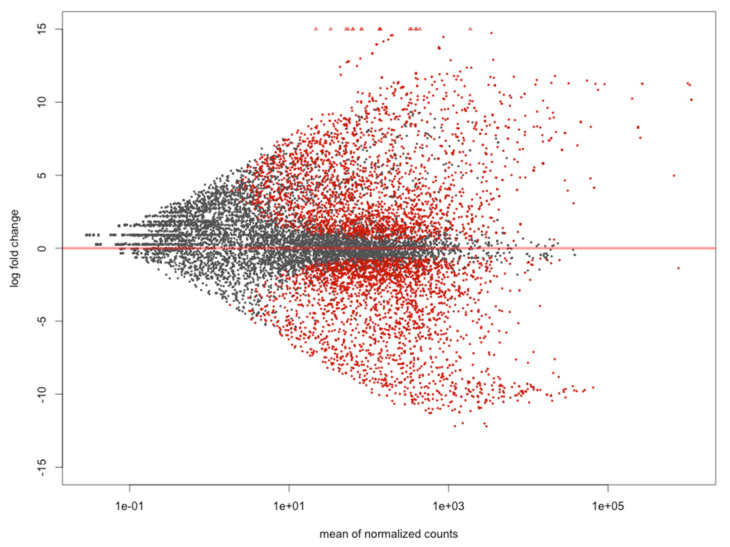
Differentially Expressed Genes (DEGs) from the comparison between unhealthy and healthy conditions after White Spot Syndrome Virus (WSSV) exposure in *Litopenaeus vannamei*. Significantly DEGs are represented by red dots (False Discovery Rate (FDR) < 0.05) and Log2FC < −0.5 for healthy and Log2FC > 0.5 for unhealthy animals. Points with negative values are up-regulated genes for healthy shrimps and those with positive values for unhealthy shrimps.

For the comparison healthy *versus* unhealthy we found 5939 DEGs (FDR < 0.05), with 2782 (47%) (log2FC < −0.5) being up-regulated in healthy (He), and 3157 (53%) (log2FC > 0.5) in unhealthy (Un) shrimps (Figure 5). Here, the up-regulated genes for healthy shrimps were down-regulated for unhealthy shrimps, and vice versa. Among the DEGs related to immune and stress responses in arthropods, we found 15% and 9% hits for healthy and unhealthy individuals, respectively (Figure 2). The most up-regulated genes in healthy shrimps were mostly those previously mentioned for hepatopancreas, in addition to inhibitor of apoptosis 2, whilst resilin, cuticle, and HSPs were the most induced in the unhealthy ones (Figure 3 and Figure 4).

**Figure 6:**
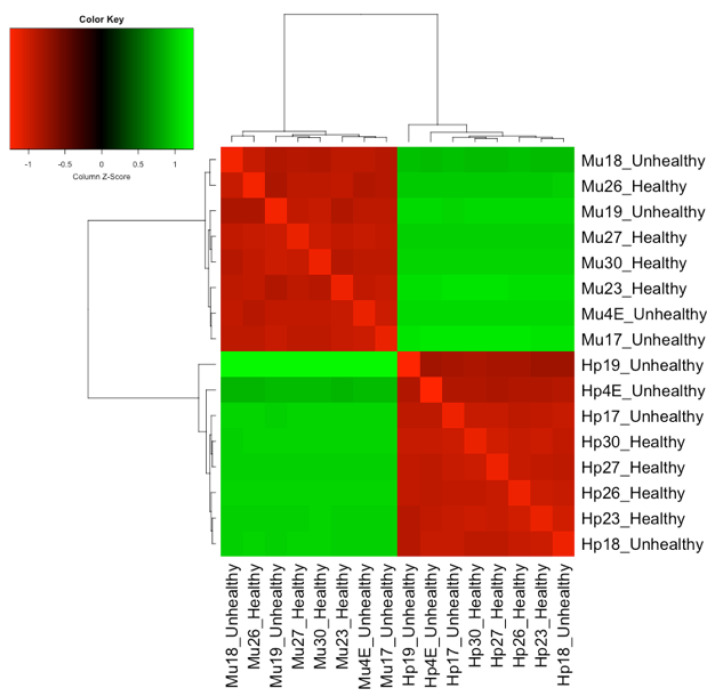
Distance between eight healthy and eight unhealthy paired samples considering the tissue effect, based on the counts per million baseMean data. Note the hepatopancreas (Hp) and muscle (Mu) of *Litopenaeus vannamei* samples separated into different groups with greater Euclidian distances. Regarding the unhealthy and healthy conditions after WSSV (White Spot Syndrome Virus) exposure, the samples from healthy shrimps are grouped only for the hepatopancreas. The bright green color indicates the highest dissimilar gene expression profiles, evidencing samples with greater Euclidean distance between them. Hepatopancreas shows greater heterogeneity between the expression profiles of healthy and unhealthy animals when compared to muscle. Samples 23, 26, 27, and 30 are from healthy animals and 4, 17, 18, and 19 from unhealthy.

When the samples from healthy and unhealthy shrimps were compared, considering the tissue effect, the hepatopancreas and muscle samples remained separated, showing different expression patterns, as expected. Regarding the condition, we observed the distribution of healthy samples gathered only in hepatopancreas, evidencing a more participating and efficient response of this organ in healthy shrimps exposed to WSSV. On the other hand, healthy and unhealthy samples were presented mixed, in contrast to hepatopancreas and muscle samples, which were clustered separately. This suggests that the difference in expression pattern observed between not-infected and infected animals is due to tissue effect, reinforcing the determinant role of tissues in healthy and unhealthy distinct expression profiles (Figure 6).

In functional GO enrichment analysis, 120 and 103 terms were identified as enriched (FDR < 0.1) for healthy and unhealthy shrimps, respectively (Table 1). The KEGG functional annotation showed the same pathways with the greater number of KO terms (KEGG Orthology) for both comparisons: (i) protein processing in endoplasmatic reticulum; (ii) Ras/Mitogen Activated Protein Kinase (MAPK) signaling pathway; (iii) endocytosis, lysosome, peroxisome, and apoptosis; and (iv) Toll and Imd signaling pathway (Figure S6 and Table S8).

### 3.3. Common Genes between Tissues and Conditions

**Figure 7:**
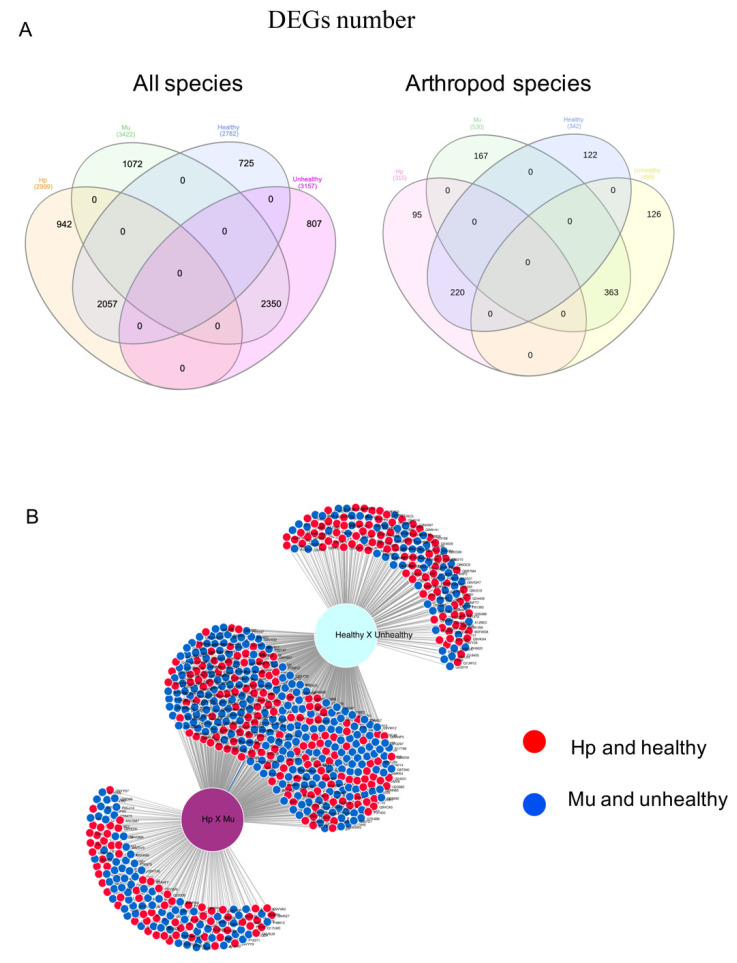
The union of Differentially Expressed Genes (DEGs) identified for hepatopancreas (Hp) and muscle (Mu) tissues and healthy and unhealthy conditions after WSSV (White Spot Syndrome Virus) exposure in *Litopenaeus vannamei*. (**A**) The numbers are shown for BLASTx hits in all species (left) and arthropod species (right). The Venn diagrams were drawn in InteractVenn [45] website (http://www.interactivenn.net). (**B**) The representation of DEGs in arthropod species. In the central area of the figure, the genes expressed simultaneously in hepatopancreas and healthy shrimps are represented by red circles and the genes expressed simultaneously in muscle and unhealthy shrimps by blue circles. Note that there are no genes in common to muscle and healthy, and hepatopancreas and unhealthy shrimps; otherwise, there would be circles highlighted in a third color in the central part of the figure. Image drawn using Di Venn [46] website (https://divenn.noble.org).

In order to detect which tissue were more active in the response against WSSV in the conditions infected or not-infected, we crossed the DEGs identified in both tissues (hepatopancreas and muscle) and conditions (healthy and unhealthy). We identified 3470 unique simultaneously differentially expressed genes between one tissue (hepatopancreas and muscle) and one condition (healthy and unhealthy), considering all BLASTx hits species. From these, we were able to find common genes only between (i) hepatopancreas and healthy shrimps and (ii) muscle and unhealthy shrimps (Table S4). As expected, the same pattern was observed when considering only the hits in arthropod species, with 363 (62%) DEGs simultaneously expressed in the muscle of infected shrimps and 220 (38%) expressed in the hepatopancreas of not-infected shrimps (Figure 7). 

Among the most induced genes in the hepatopancreas of healthy shrimps were those related to immunity and stress, as crustacyanin, β -1,3-glucan-binding, peritrophin, and trypsin. Conversely, in the muscle of unhealthy shrimps, we found those related to muscle development and maintenance, such as actin, myosin, and troponin, followed by the ones related to chitin metabolism (resilin and cuticle proteins) and immunity and stress (heat shock, pro-clotting, serine proteases, and lectin) (Figure 4).

### 3.4. Protein-Protein Interaction (PPI) Networks for the Most Up-Regulated Genes

After the identification of the most expressed genes for each tissue and condition, the main protein-protein interaction (PPI) networks were constructed for the BLASTx hits in arthropods only. Using the STRING database as a reference, we identified 120 and 118 protein pairs for hepatopancreas and muscle tissues, and for healthy and unhealthy conditions, respectively. Focusing on proteins participating in immune or stress-related pathways, the main protein-protein interactions were: Tyrosine-protein kinase (Src42A) (pp) Nuclear factor NF-kappa-B p110 subunit (Rel), Caspase Dronc (Dronc) (pp) Rel, and Dronc (pp) Death-associated inhibitor of apoptosis 2 (Diap2) (Table S9). *Src42A* and *Dronc* were found simultaneously up-regulated in the hepatopancreas and healthy shrimps, with many of these proteins interacting with each other (Figure S7 and Table S9). On the other hand, Rel and *Diasp2/IAP* were only up-regulated in hepatopancreas and in healthy shrimps, respectively. All these genes are involved in cellular events related to apoptosis, innate immunity and/or response to stress/stimuli and the interactions presented high reliability (score) and strong interaction (EdgeBetweenness) values. In unhealthy shrimp muscle, we observed the PPIs mostly between HSP proteins (Table S9).

## 4. Discussion

This study analyzed differentially expressed genes in *L. vannamei* with the aim of understanding the shrimp immune response to WSSV. For this purpose, we evaluated the transcriptome from hepatopancreas and muscle of *L. vannamei* under two conditions after exposure to WSSV: symptomatic infected (unhealthy) and asymptomatic not-infected (healthy). The hepatopancreas was chosen due to its role in shrimp immunity, as producing many defense proteins. Besides, muscle has mesodermal origin and are among the tissues affected by the syndrome [27]. The transcripts were mapped against the recent described *L. vannamei* genome [32], making this study the first report in literature in which RNA-seq data from *L. vannamei* shrimps exposed to WSSV used the species genome as a reference, increasing the reliability of the results. Another noteworthy difference in our work was the contagion method used. Contrasting with some of the most recent *L. vannamei* transcriptome studies involving WSSV infection [5,32], here we used SPF shrimps naturally exposed to the WSSV under standard husbandry conditions in land tanks in a Brazilian shrimp farm with high incidence of the virus. Such contagion strategy was adopted in order to promote exactly the same contagion conditions observed in aquaculture farms. Thus, shrimps were infected by the contact with the virus transported over the water [47,48], as usually happens in a farming environment, instead of virus injections, as commonly reported in the literature [49,50,51]. 

Although all PLs used in our assay came from a single batch and have been under a constant water recirculating system, some shrimps were not infected with the virus and did not display any WSSV clinical signs. In contrast, other individuals were infected and drastically affected, demonstrating several clinical signs, as markedly features resulting from WSSV infection [52]. Despite we did not measure the viral concentration at the pond water, qPCR tests were performed before and after the exposure to WSSV, and the results showed that all PLs initially used in the experiment were WSSV-negative. Moreover, for the sampling, shrimps were locally collected in a single fishing, and then submitted to morphological and qPCR analyses. Both diagnoses confirmed no viral charge in the asymptomatic sampled individuals, whilst symptomatic shrimps were WSSV-positive (Table S1). We did not find any WSSV-positive asymptomatic shrimp. 

After mapping our transcriptome data against the WSSV genome, we could detect correspondence between shrimp reads and WSSV gene copies only in the unhealthy group, reinforcing that healthy shrimps were WSSV-free. Despite the fact that we did not find virus copies for two WSSV-positive samples, these results can be observed in shrimps with lower viral load, as previously reported in *F. chinensis*, in which WSSV sequence copies were identified only in a group with a higher viral load [53]. The immediate-early (IE) and ribonucleotide reductase small subunit viral genes were up-regulated in WSSV-positive shrimps. It is known that these two proteins act in enabling the virus entrance and replication in host cells. IE proteins relies only on host proteins and a previous protein synthesis is not required. They are vital for virus life cycle and leads to inactivation of host immune defenses [54,55]. The ribonuclease reductase participates in the conversion of ribonucleotides to deoxyribonucleotides, providing replication advantages to the virus [56,57]. These two proteins are shown to be determinant in WSSV infection [23,58].

The innate immune system in shrimp is basically formed by humoral and cellular immune responses, both with action against WSSV [59,60]. The humoral responses are usually the first ones to be triggered by contact with the pathogen, when pathogen-associated molecular patterns (PAMPs), such as viral antigens, are recognized by the pattern recognition proteins (PRPs) in cell membranes [61]. This interaction leads to the activation of immune pathways, such as Imd, with the later release of antimicrobial peptides (AMPs) in response to pathogens [9,62]. In our study, we could only detect up-regulated genes being expressed simultaneously in the hepatopancreas of healthy shrimps, and in muscle of unhealthy shrimps, indicating the determinant role of hepatopancreas in the shrimp immune response against WSSV [62,63] and suggesting that genes up-regulated in this organ and in healthy shrimps are among the central ones in keeping shrimps WSSV free. In addition, several immune related genes were identified up-regulated in the hepatopancreas and/or in healthy shrimps, such as (AMPs), anti-lipopolysaccharides factors (ALFs), and Pattern Recognition Proteins (PRPs). These results suggest that the not-infected shrimps may have an efficient immune system that allows the organism to effectively eliminate or prevent the virus entrance in the body, as discussed from now onward.

Crustacyanins are unique to crustaceans and act in carapace color. In addition, these proteins are known for acting in defense responses against pathogens, including WSSV [22]. Here, we found crustacyanin subunit A2 highly up-regulated only in the hepatopancreas of healthy shrimps. The presence of the type A2 strongly expressed in healthy shrimps may indicate some possible function related to organism protection, during the exposure to the WSSV, agreeing with previous studies about the protection effect assigned to crustacyanin A2 in *L. vannamei* [9,64]. We also found lysozyme gene up-regulated in the hepatopancreas of healthy shrimps after the exposure to WSSV. Lysozymes are known by their protection action against pathogens, as WSSV [65]. In *Litopenaeus styrilostris,* higher survival rates and a decreased viral load were observed in shrimps first injected with lysozyme and then challenged to WSSV [66]. As reported by Mai et al. [66], shrimps previously treated with lysozymes did not become ill after the following contact with WSSV. Therefore, our data indicate that lysozyme is also active in shrimps exposed to the virus and may have a potential role in preventing the WSSV contagion in *L. vannamei*, as well. 

Beyond this novelty, we report, for the first time, the proPO3 gene up-regulated in the hepatopancreas of healthy *L. vannamei* shrimps. Prophenoloxidases (proPO) genes are known to be involved in the melanization and isoforms 1 and 2 were previously reported down regulated in the hemocytes of *L. vannamei* challenged with WSSV [13,67]. Nevertheless, a possible role for *proPO3* in WSSV protection has not been reported in shrimp to date. Melanization is known as an important pathway in shrimp immune responses [51,60]. In short, the melanization cascade is activated by the proPO enzymes, producing cytotoxic metabolites, as melanin, to help in pathogen encapsulation [60,68]. PRPs recognize the PAMPs in cell membranes and the serine proteinases act as melanization inhibitors, to control the melanization side effects, as apoptosis [49,69]. Melanization activity against WSSV has already been reported in *L. vannamei* [13] and *Fenneropenaeus chinensis* [70], though its mechanism of action is not fully known. 

Our findings also bring β-1,3-glucan-binding protein *(bGBP)* with increased expression in shrimps exposed to WSSV as novel information, given we can detect the *bGBP* expression response for the first time after WSSV contact in *L. vannamei*, via waterborne route [47,48], not injection. Here, we found *bGBP* up-regulated in the hepatopancreas of healthy shrimps. As previously reported, the *bGBP* was found induced in the hepatopancreas of *M. japonicus* [62], *P. styrilostris* [71], and *F. chinensis* [72] after contact with WSSV. The treatment with dsRNA *LvbGBP* showed a 100% death rate 72 h after the WSSV injection when compared to 50% mortality in control group after the same time [73]. We also detected genes from the serine protease family, such as trypsin and chymotrypsin, to be up-regulated in the hepatopancreas of healthy shrimps. They are known as participants in many cellular events (e.g., hemolymph clotting) and are known to be related to immunity in shrimp [74,75]. Regarding *L. vannamei*, the studies rely on White Spot virus protein injections only, resulting in increased expression levels of trypsin and chymotrypsin genes in the gills after the contact with the virus proteins [76], pointing out potential candidate genes to be applied in white spot control. 

Genes from Imd pathway, such as *Rel* and *ankyrin*, were also found up-regulated in the hepatopancreas of healthy *L. vannamei* shrimps, suggesting their role in protection against WSSV contagion. The Imd signaling pathway triggers the NF-κB pathway in arthropods, mostly by contact with PAMPs. All proteins from NF-κB pathway own a Relish *(Rel)* homology domain, responsible for the production of AMPs that act in immune responses [59]. The Imd pathway is also regulated by the nuclear factor Ankyrin, acting together or downstream of the transcription factor Relish [77,78]. For WSSV, Imd pathway is suggested to be used by the virus to enable its multiplication and infection in the host [59]. *Rel* was reported overexpressed in the gills of *L. vannamei* challenged for White Spot [10] and *ankyrin* was observed induced in the hepatopancreas of *L. vannamei*, but with no role mentioned in WSSV protection [77]. Here, we bring, for the first time, both transcription and nuclear factors from Imd pathway up-regulated in the hepatopancreas of healthy shrimps exposed to the WSSV but WSSV-free, contributing to a more clarifying role of Imd in the prevention of infection. 

The Relish factor needs to interact with a caspase enzyme to become active and direct regulate the AMPs synthesis [60]. We found that expression of caspase genes increased in unhealthy shrimps, suggesting that WSSV triggers cell death [79] and evidencing the importance of the apoptosis inhibitors (IAP) in the balance between virus and host death. *L. vannamei* IAP2 *(LvIAP2)* seems to be essential for shrimp survival during WSSV infection, since, when *LvIAP2* was silenced by interference RNA, all infected *L. vannamei* shrimps died in 48 h [78], potentially linking the *LvIAP2* action with AMPs regulation. In our RNA-seq data, we found the Dronc-like caspase gene up-regulated in hepatopancreas of healthy shrimps, while *LvIAP2* was induced only in healthy shrimps, regardless of the tissue, highlighting the determinant importance of *LvIAP2* in the shrimp survival also after WSSV exposure, without virus injection. 

A relevant finding in our data that reinforces the Imd participation in WSSV defense was the strong PPI observed between Relish and Dronc caspase (Table S9 and Figure S7). A strong protein interaction Dronc (pp) Diap2/LvIAP2 was detected, pointing out apoptosis inhibitors as mandatory for cell death control and host survival rates. Caspase inhibitors have already been reported as facilitators of the WSSV infection by impairing cell death and enabling the virus to spread through the body [50,80,81], but, intriguingly, our findings suggest a novelty. It is plausible that cell death in the hepatopancreas of not-infected shrimps provides potential protection to WSSV, as concluded from the apoptosis related genes we found overexpressed in the hepatopancreas of healthy shrimps *(*Figure 4). In this case, the caspase inhibitors, mainly *LvIAP2*, turn out to be potential candidates to control the mortality rates in shrimp farming threatened by WSSV.

Curiously, we also observed a chitin metabolism-related gene, peritrophin-1 or A, among the immune-related genes expressed in the hepatopancreas of healthy shrimps. Peritrophin may interact with many proteins from the White Spot virus envelope and mediate its infection in *Exopalaemon carinicauda* [82] and *L. vannamei* [83,84] after virus injection. Studies have already reported that WSSV may have access to shrimp body through oral or waterborne routes [47,48]. Digestive tract structures constitute an efficient barrier against pathogens mainly due to a cuticle layer and a digestive epithelium, with pores smaller than the virus size. Here, we found the peritrophin gene strongly up-regulated in the hepatopancreas of healthy *L. vannamei* shrimps, which is an unexpected result, since the previous studies reported peritrophin role in facilitating the virus entrance in the body. In our study, healthy shrimps were exposed to the virus but remained WSSV-free. 

Overall, we observed the much greater number of GO enriched terms for hepatopancreas when compared to muscle (Table S7), suggesting a scenario where the organism is possibly coping with high levels of stress, followed by an effort to manage successfully the pathogen disturbances, through cellular events, such as virus recognition, melanization, and apoptosis. The same can be noted when considering the main pathways identified in KEGG mapping involved in response to stimuli, endocytosis, peroxisome, apoptosis, and Imd signaling, observed only in healthy shrimps. On the other hand, some genes were also up-regulated in the muscle of unhealthy shrimps not coping efficiently against WSSV infection. 

In crustaceans, most of the proteins involved in immunity are produced and stored in the hemocytes before they are released into the hemolymph [9,60], justifying the detection of immune proteins in muscle, as well. However, according to our data, pro-resilin, cuticle proteins, hemolymph clottable, proclotting enzyme, and many HSPs genes differentially expressed in unhealthy condition (Figure 4) cannot be pointed out as good candidates in WSSV control since they were up-regulated in unhealthy infected shrimps with strong clinical signs. In addition, the cuticle, chitinase, and pro-resilin genes strongly up-regulated in unhealthy shrimps suggest a inhibition in molt process, with the majority of energy being required for immune defense [85].

## 5. Conclusions

RNA-seq technology and analysis has evolved enormously in recent years, and it is now widely accepted as a benchmark of gene expression quantification [86,87]. With the purpose of a better comprehension of the genes involved in the protection against WSSV in *L. vannamei* organism, we sought for immune responses triggered in shrimps exposed to WSSV but that remained WSSV-free. Several genes are described here, and some of them, as peritrophin and proPO3, are highlighted as up-regulated for the first time in *L. vannamei* exposed to WSSV. All the protein-coding genes presented here up-regulated in the hepatopancreas of healthy shrimps are good candidates to be used for further functional analyses, such as RNAi and qPCR, and other approaches that consider the development of efficient prophylactic management strategies for WSSV control in shrimp production. Overall, this work contributes to a better understanding of the molecular response to WSSV exposure in penaeids, identifying the DEGs in *L. vannamei,* their respective coding proteins, the immune responses triggered in the battle against the pathogen, and the differential responses came from hepatopancreas in shrimps not-infected and infected by WSSV. However, we argue that studies to elucidate the mechanism of action and how these proteins interact with each other against the virus are still needed.

## Figures and Tables

**Table 1:** Summary of the Gene Ontology (GO), showing Biological Process (BP), Molecular Function (MF), and Cell Component (CC) terms (FDR < 0.1) from differentially expressed genes between healthy and unhealthy *Litopenaeus vannamei* exposed to WSSV (White Spot Syndrome Virus), and resulting enriched pathways.

GO ID	GO Category	Tissue	Ajusted *p-Value*	No. Genes	GO Term
GO:0044281	BP	Healthy	4.21 × 10^−8^	305	Small molecule metabolic process
GO:0009117	BP	Healthy	1.6 × 10^−4^	64	Nucleotide metabolic process
GO:0045454	BP	Healthy	2.0 × 10^−3^	18	Cell redox homeostasis
GO:0070062	CC	Healthy	3.0 × 10^−4^	183	Extracellular exosome
GO:0072546	CC	Healthy	4.0 × 10^−3^	7	ER membrane protein complex
GO:0043492	MF	Healthy	1.5 × 10^−4^	27	ATPase activity, coupled to movement of substances
GO:0001872	MF	Healthy	7.1 × 10^−3^	4	(1-3)-β-D-glucan binding
GO:1902903	BP	Unhealthy	1.0 × 10^−4^	36	Regulation of supramolecular fiber organization
GO:0044042	BP	Unhealthy	1.0 × 10^−4^	17	Glucan metabolic process
GO:0035556	BP	Unhealthy	1.0 × 10^−4^	118	Intracellular signal transduction
GO:0001558	BP	Unhealthy	6.0 × 10^−4^	44	Regulation of cell growth
GO:0030018	CC	Unhealthy	2.2 × 10^−3^	26	Z disc
GO:0044430	CC	Unhealthy	3.1 × 10^−3^	142	Cytoskeletal part
GO:0042383	CC	Unhealthy	3.7 × 10^−3^	23	Sarcolemma
GO:0051015	MF	Unhealthy	3.1 × 10^−3^	17	Actin filament binding
GO:0008092	MF	Unhealthy	3.6 × 10^−3^	61	Cytoskeletal protein binding

## Supplementary Materials

The following are available online at https://www.mdpi.com/2073-4425/11/7/805/s1, Figure S1: Sampling scheme of the eight WSSV-exposed *Litopenaeus vannamei* shrimps used in hepatopancreas (Hp) x muscle (Mu) and healthy x unhealthy comparisons. The hepatopancreas and muscle tissues of healthy and unhealthy animals were sequenced (*n* = 8, 16 cDNA libraries in total). Figure S2: Union of all Differentially Expressed Genes (DEGs) that returned hits in SwissProt, Gene Ontology (GO), Kyoto Encyclopedia of Genes and Genomes (KEGG), and Clusters of Orthologous Groups (COG) databases. Figure S3: Differentially Expressed Genes (DEGs) resulting from the comparison between hepatopancreas and muscle in *Litopenaeus vannamei*. Significantly DEGs are represented by red dots (FDR < 0.05). The negative points are up-regulated in hepatopancreas (negative log2FC values) and the positive in muscle (positive log2FC values). Figure S4. Euclidean distances between the eight hepatopancreas (Hp) and eight muscle (Mu) samples paired, based on normalized counts per million values baseMean. Note the large difference between the gene expression profiles of Hp and Mu samples (two green quadrants) in *Litopenaeus vannamei*. The color intensity indicates their expression profiles heterogeneity. Here, the green color represents the greater distances and very dissimilar values of gene expression between the pair of samples. The numbers 4, 17, 18, 19, 23, 26, 27, and 30 after the Hp and Mu names represent the replicates. Samples 23, 26, 27, and 30 are from healthy animals and 4, 17, 18, and 19 from unhealthy individuals exposed to the White Spot Syndrome Virus (WSSV). Figure S5: Top 10 GO Biological Processes (BP), Molecular Function (MF) and Cellular Component (CC) identified in hepatopancreas (Hp) x muscle (Mu) and healthy x unhealthy comparisons for *Litopenaeus vannamei* shrimps exposed to the White Spot Syndrome Virus (WSSV). Figure S6: The most mapped KEGG (Kyoto Encyclopedia of Genes) pathways according to KOs identified in hepatopancreas (Hp) x muscle (Mu) and healthy x unhealthy shrimp comparisons for *Litopenaeus vannamei* shrimps exposed to the White Spot Syndrome Virus (WSSV). Figure S7. Network of protein-protein interactions as visualized in Cytoscape. The most up-regulated genes and their respective proteins are represented as nodes (circles) for (A) hepatopancreas x muscle (Hp x Mu); and (B) healthy x unhealthy comparisons for *Litopenaeus vannamei* shrimps exposed to the White Spot Syndrome Virus (WSSV). Only the protein-protein interactions (PPIs) with higher values of score and EdgeBetweenness are represented. The nodes (circles) sizes are proportional to the number of interactions one protein makes with others in the network (degree). The connecting lines thickness shows the strength of the binding between two proteins in the network. The nodes (circles) highlighted in yellow (A) and (B) represent the genes up-regulated in Hp and Healthy shrimps, respectively. The genes circled are involved in phagosome pathway (green), immune system process (red), and response to stress (blue). Table S1: qPCR for WSSV (White Spot Syndrome Virus) diagnosis results in the WSSV-exposed *Litopenaeus vannamei* shrimps. The threshold cycle (Ct) and virus load are shown. Table S2: The up-regulated genes from *Litopenaeus vannamei* shrimps exposed to the White Spot Syndrome Virus (WSSV) used as input for PPI analysis in STRING database. The locus name, log2FC, and Uniprot ID are shown. Table S3: Total fragments originated from the Illumina sequencing, after filtering in the Seqyclean and mapping for the *Litopenaeus vannamei* samples exposed to the White Spot Syndrome Virus (WSSV). Table S4: Significant Differentially Expressed Genes (DEGs) (FDR < 0.05) resulted from comparison between hepatopancreas (Hp) versus muscle (Mu) tissues (Hp x Mu); healthy (He) versus unhealthy (Un) conditions, considering the tissue effect in a multi-factor analysis (He x Un); muscle of healthy (MuHe) versus muscle of unhealthy (MuUn) shrimps (MuHe x MuUn); and hepatopancreas of healthy (HpHe) versus hepatopancreas of unhealthy (HeUn) ones (HpHe x HeUn). In addition, the DEGs induced simultaneously in the Hp and healthy and in the muscle and unhealthy *Litopenaeus vannamei* shrimps and the DEGs unique to the four comparisons are also presented. The log2FC values and their respective p-values and adjusted p-values (padj or FDR) are shown. Table S5: *Litopenaeus vannamei* transcriptome mapped against the WSSV genome results. Number of shrimp transcriptome reads mapped (top), the number of WSSV gene copies mapped in the shrimp transcriptome (middle), and the four differentially expressed WSSV genes (bottom). Table S6: All unique unigenes from hepatopancreas x muscle (Hp x Mu) and healthy x unhealthy (He x Un) comparisons from *Litopenaeus vannamei* that returned hits in Gene Ontology (GO). The Biological Process (BP), Molecular Function (MF), and Cellular Component (CC) terms are shown. Table S7. Summary of Gene Ontology (GO) for the *Litopenaeus vannamei* transcriptome, describing Biological Process (BP), Molecular Function (MF), and Cell Component (CC) terms (FDR < 0.1) from differentially expressed genes between hepatopancreas (Hp) and muscle (Mu), as well as their enriched pathways. Table S8: All unique unigenes from hepatopancreas x muscle (Hp x Mu) and healthy x unhealthy (He x Un) comparisons for the *Litopenaeus vannamei* transcriptome that returned hits in Kyoto Encyclopedia of Genes and Genomes KEGG. The unique KEGG Orthology (KO) numbers are shown. Table S9. The protein-protein interactions (PPI) with the highest values of Score and EdgeBetweenness. Here, we considered only the Differentially Expressed Genes (DEGs) with log2FC ≥ 3 (absolute value) and genes coding for proteins related to immunity in the *Litopenaeus vannamei* transcriptome.
