# Supplementary material for: Shedding the Light on Litopenaeus vannamei Differential Muscle and Hepatopancreas Immune Responses in White Spot Syndrome Virus (WSSV) Exposure
Source: Genes (Basel). 2020 Jul 16;11(7):805. doi: 10.3390/genes11070805 (PMC7397224; doi:10.3390/genes11070805)
Supplement: Supplementary file 1 [file genes-11-00805-s001.zip › Suplementary Material_Genes/S1_Table.docx]

| **Illumina reads** | | | | |
| --- | --- | --- | --- | --- |
|  | **WSSV-exposed shrimp** | | | |
| **Tissue** | **Muscle**  **(8 libraries)** | | **Hepatopancreas**  **(8 libraries)** | |
| **Condition** | **Healthy** | **Unhealthy** | **Healthy** | **Unhealthy** |
| Paired-end reads | 248.795.146 | 235.537.380 | 266.191.030 | 233.223.996 |
| Average paired-end reads/ library (millions) | 62.1 | 58.8 | 66.5 | 58.3 |
| Standard deviation | 5.585.147 | 4.580.309 | 6.611.724 | 6.768.318 |
| Total by tissue | 484.332.526 | | 499.415.026 | |
| **Total** | **983.747.552** | | | |
| **After SeqyClean filtering** | | | | |
| Paired-end reads | 189,228,688 | 180,458,256 | 207,789,948 | 185,460,392 |
| Average paired-end reads/ library (millions) | 48,8 | 41,7 | 51,9 | 45,1 |
| Standard deviation | 3,591,730 | 2,843,996 | 4,347,555 | 4,735,157 |
| Total by tissue | 369,686,944 | | 393,250,340 | |
| **Total** | 762,937,284 (77%) | | | |
| **After mapping** | | | | |
| Paired-end reads | 138,217,662 | 128,282,688 | 148,815,842 | 130,342,298 |
| Total by tissue | 266,500,350 | | 279,158,140 | |
| **Total mapped** | 545,658,490 (71,5%) | | | |
