# Supplementary figures and images for: Shedding the Light on Litopenaeus vannamei Differential Muscle and Hepatopancreas Immune Responses in White Spot Syndrome Virus (WSSV) Exposure
Source: Genes (Basel). 2020 Jul 16;11(7):805. doi: 10.3390/genes11070805 (PMC7397224; doi:10.3390/genes11070805)

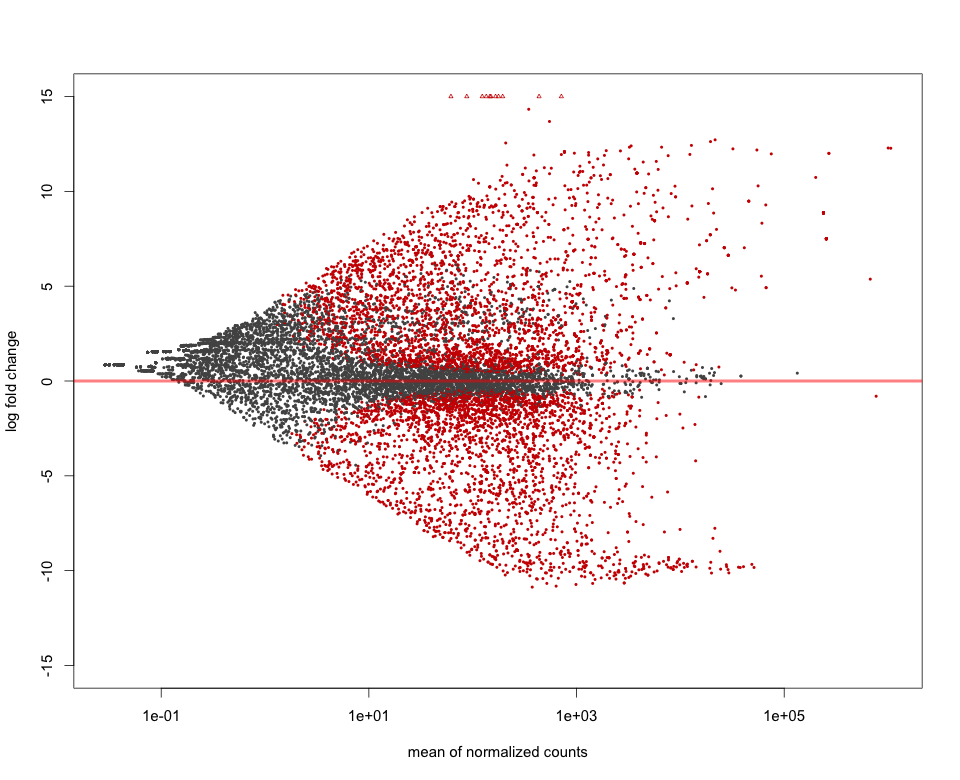

Supplement: Supplementary file 1 [file genes-11-00805-s001.zip › Suplementary Material_Genes/S3_Figure.tiff]

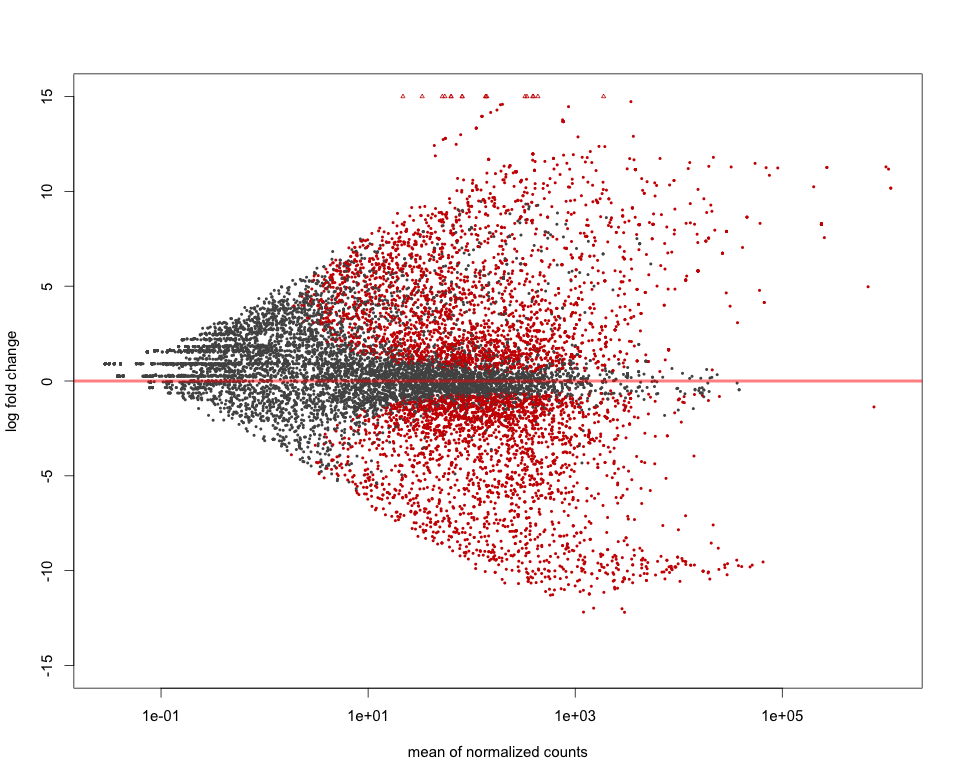

Supplement: Supplementary file 1 [file genes-11-00805-s001.zip › Suplementary Material_Genes/S5_Figure.tiff]
